# Assessing nursing students’ palliative care training needs and profiles: a cross-sectional study using K-means clustering

**DOI:** 10.1186/s12912-025-03797-0

**Published:** 2025-10-06

**Authors:** Liu Yang, Lanxin Zhang, Bingjie Long, Tong Zhu, Mei Chen, Simon Ching Lam, Renli Deng

**Affiliations:** 1 Department of Emergency, Affiliated Hospital of Zunyi Medical University, No.149, Zunyi, Guizhou, China; 2 School of Nursing, Zunyi Medical University, Zunyi, China; 3 School of Nursing, Tung Wah College, 31 Wylie Road, Homantin, Kowloon, Hong Kong SAR China

**Keywords:** Education, K-means clustering, Nursing students, Palliative care, Training needs

## Abstract

**Background:**

Palliative care is crucial in nursing, yet nursing students often lack adequate training, compromising their ability to provide holistic end-of-life care. Despite growing awareness, research on specific training needs remains limited.

**Aim:**

This study aimed to assess and classify the training needs of nursing students in palliative care education by using K-means clustering.

**Design:**

A cross-sectional study.

**Methods:**

Data were collected in March 2023 from nursing students at a medical university in southwestern China through an online questionnaire. The questionnaire included demographics and the Nurses Receiving In-service Palliative Care Education Needs Scale. Subsequently, K-means clustering was used to analyze the collected data, aiming to identify distinct profiles of nursing students with similar palliative care training needs.

**Results:**

A total of 695 nursing students participated in the study, with a mean age of 22.40 years and 87.6% being female. Most of the students lacked experience in terminal care (80.4%) and nontextbook palliative care knowledge (74.4%). Nursing students prioritized training needs in ethical issues and teamwork (mean score: 3.71), communication and consultation (mean score: 3.69), and managing symptoms and pain relief (mean score: 3.69), while cultural and spiritual considerations ranked lowest (3.41). K-means clustering identified two distinct profiles: high-level needs (*n* = 393) and moderate-level needs (*n* = 302). High-need students reported significantly lower exposure to nontextbook palliative care knowledge compared to moderate-need students (*p* < 0.001).

**Conclusion:**

Nursing students exhibit high-level needs of palliative care training, particularly in ethical issues and teamwork, communication and counseling, and handling of symptoms and pain relief. K-means clustering identified two profiles: high-level needs and moderate-level needs groups, differentiated by nontextbook learning exposure. Nursing educators should urgently consider redesigning palliative care training curricula, addressing resource accessibility gaps, and tailoring programs to cluster-specific needs to improve palliative care preparedness.

## Introduction

Palliative care is essential to quality nursing practice, focusing on optimizing quality of life for patients with serious illnesses through holistic support for physical, psychological, social, and spiritual needs [1–3]. By emphasizing early needs identification and patient-centered interventions, palliative care addresses complex challenges such as pain, dyspnea, and fatigue, as well as psychological distress, social challenges (especially those related to caregiving arrangements and financial toxicity), and spiritual concerns [2]. Nursing students, as future clinical practitioners, are key palliative care providers whose competencies directly shape patient care quality and experiences [4]. Palliative care requires nursing personnel to master core competencies, including communication, pain management, and emotional support, which nursing students must acquire through specialized training [5, 6]. However, current nursing curricula often lack targeted training, resulting in significant knowledge gaps that compromise care quality and patient satisfaction [7]. Consequently, strengthening palliative care education to enhance students’ clinical preparedness represents an urgent priority in nursing education.

Despite increasing global recognition of palliative care’s importance in nursing education [8, 9], research on the specific training needs of nursing students in this field remains limited, particularly in China. While existing studies have assessed students’ overall knowledge and attitudes toward palliative care globally [10–12], few have explored their training needs in palliative care [13, 14] or death education [15]. However, none have systematically identified homogenous subgroups of students with shared training needs (e.g., groups characterized by similar knowledge gaps, clinical exposure levels, or learning preferences) to enable targeted educational interventions. For instance, students lacking practical experience in end-of-life care may require simulation-based training, whereas those with moderate theoretical knowledge might benefit from advanced communication skills workshops. This gap highlights the need for a more nuanced understanding of training needs to tailor educational strategies effectively. To address this gap, our study uses K-means clustering to identify distinct subgroups of Chinese nursing students with homogeneous palliative care training needs, guiding the development of tailored educational strategies for each subgroup.

## Background

Palliative care is a person-centered, interdisciplinary approach that enhances quality of life for patients with life-limiting conditions and their families by addressing physical, psychological, social, and spiritual needs across all ages and disease stages [2]. Registered nurses must demonstrate comprehensive competencies in palliative care, including clinical expertise in managing symptoms (e.g., pain, dyspnea, nausea, fatigue, anorexia) and holistic capabilities to address patients’ and families’ multidimensional needs across physical, psychological, social, and spiritual domains. These competencies extend to advanced practice skills, such as formulating individualized care plans, coordinating multidisciplinary teams, and providing patient/family education throughout the illness trajectory [16, 17]. These competencies align with the American Association of Colleges of Nursing’s Essentials, which mandate that nursing students graduate with foundational knowledge and skills in palliative care, including symptom management, communication, ethical decision-making, and interprofessional collaboration, to meet the complex needs of patients with serious illnesses [18].

Research evidence indicates that health care professionals, including practitioners and students under training, generally lack fundamental knowledge and skills in palliative care; as a result, practical gaps exist in the clinical and psychological management of severe patients [14, 19, 20]. A review article analyzing 26 primary research papers, involving 1586 nursing students, revealed that the absence or lack of an in-depth and holistic curriculum on palliative and end-of-life care had left many nursing students feeling inadequate in communicating with and caring for dying patients and their families, as well as coping with patient death [7]. However, the content and quality of palliative care training within the current nursing education system have notable disparities [21], and nursing students’ knowledge of palliative care is influenced by various factors, including gender, religious beliefs, prior education in palliative care, experience in caring for dying patients, and personal bereavement experiences [12]. Consequently, the training needs exhibit diverse and individualized characteristics.

For instance, a comprehensive review of 13 studies conducted across 26 countries revealed substantial knowledge demands in key areas of palliative care, such as its principles and philosophy, pain and symptom management, and psychosocial/spiritual care. These findings suggest that nursing students across different cultural and educational backgrounds share common needs in their training for palliative care [19]. Saudi [22] and Athenian [10] nursing students demonstrated strong needs for understanding palliative care principles and philosophy, pain and symptom management, and psychosocial/spiritual care. Additionally, research on Chinese nursing students has shown that they have significant demands for specialized knowledge and skills in symptom management, terminal illness care, awareness of the dying process, post-mortem care, interdisciplinary team collaboration, and safeguarding the rights and interests of dying patients [13]. While nursing students may have common training needs, the intensity of these needs and the specific areas they prioritize can differ. Assessing their self-perceived needs and developing need-based profiles can both illuminate current educational status and inform personalized training program design.

## Study

### Aim

This study aimed to assess nursing students’ self-reported training needs in palliative care and to identify clinically meaningful subgroups through need profiles, thereby informing the development of more targeted and effective educational interventions.The research questions were as follows: (1) What is the level of nursing students’ training needs in palliative care education? (2) Can these training needs be classified into different profiles based on their levels or characteristics?

### Design

This study used a cross-sectional research design. The methodological procedures for this study were conducted in accordance with the Strengthening the Reporting of Observational Studies in Epidemiology guidelines for cross-sectional studies [23].

### Participants

Given that palliative care education is highly practical, the participants of this study were year 3 and year 4 full-time undergraduate students at a medical university in southwestern China (year 3 students are about to embark on clinical placements, while year 4 students are already engaged in clinical practice; the teaching arrangement for palliative care education for nursing students is presented in Table 1). All students who fulfilled the eligibility criteria were invited to participate in the study. The inclusion criteria were as follows: (1) aged 18 years or older, (2) year 3 or year 4 full-time undergraduate students, and (3) willing to participate in the study. Students with serious health or psychological issues hindering study participation or task completion were excluded. To ensure a robust and representative sample, we calculated the minimum sample size based on the guidance provided by Schönbrodt and Perugini [24]. Specifically, we aimed to achieve a true correlation of 0.15 with a power of 80% and a 5% type 1 error rate. This calculation yielded a minimum sample size of 347 participants. This approach ensures that our correlations are stable and our results are reliable, minimizing sampling error and enhancing the generalizability of our findings.

**Table 1:** Teaching arrangement of palliative care for nursing students

Grade	Teaching material	Chapter	Section
Year 1	Fundamental Nursing	End-of-life care	Hospice care
			Dying and death
			Hospice care for patients and their families
			Postmortem care
Year 2	/		
Year 3 and Year 4	Geriatric nursing	Elderly palliative care	Elderly palliative care
			Death education for the elderly
			Grief counseling for elderly family members

### Instrument

The questionnaire comprised two sections: (1) demographic characteristics and (2) the Nurses Receiving In-service Palliative Care Education Needs Scale [25].

Demographic characteristics included questions on age, gender, ethnicity, year level, religious beliefs, only child, experience in caring for terminally ill patients or their relatives, acquisition of knowledge about palliative care from nontextbook sources, and involvement in palliative care training/seminars.

The Nurses Receiving In-service Palliative Care Education Needs Scale is a self-reporting tool to assess in-service nurses’ level of palliative care training needs [25]. Ziwei et al. [13] applied the questionnaire to nursing students and established the priorities of their educational needs in the field of palliative care. The scale comprises 40 items in six dimensions, including (1) handling of symptoms and pain relief (eight items), (2) ethical issues and teamwork (eight items), (3) preparation and care before death (six items), (4) concept of palliative care (seven items), (5) communication and counseling (seven items), and (6) cultural and spiritual considerations (four items). Each item was measured using a five-point Likert scale ranging from 1 to 5, with 5 points indicating “extremely necessary,” 4 points indicating “very necessary,” 3 points indicating “somewhat necessary,” 2 points indicating “not very necessary,” and 1 point indicating “extremely unnecessary.” The total number of points ranged from 40 to 200, and the greater the number of points, the more the students felt that the stated item was training needed. The item content validity index was 0.98, the scale content validity index was 0.88, and Cronbach’s alpha was 0.982 [25].

### Data collection

Data were collected using the online survey platform Survey Star between March 13 and March 22, 2023. To maximize participation rates, the survey link was distributed through eight class counselors, who serve as administrators of official student WeChat groups. These counselors forwarded the link to their students via WeChat messages. Class counselors were chosen because they are the primary administrators of student cohorts and have frequent interactions with students, making them well-suited to facilitate the distribution of the survey and encourage participation. To ensure data quality, we implemented multiple safeguards. An electronic informed consent form at the survey outset explained the study purpose and confidentiality measures. Survey Star’s built-in IP restriction prevented duplicate submissions. A minimum completion threshold of 3 min was set to exclude rushed responses that might compromise data reliability. Attention-check items (e.g., “Please select ‘Strongly disagree’ for this item”) identified inattentive participants. Additionally, two trained researchers independently screened the submissions, excluding participants who exhibited straight-lining patterns. To minimize bias, all researchers received standardized training in objective data collection, and participants were provided with clear instructions to enhance response accuracy. All data were collected automatically, with anonymity preserved throughout the process.

### Data analysis

Statistical analyses were performed using SPSS software (version 29.0). The results are reported as descriptive statistics, including frequency, percentage (%), mean, and standard deviation (SD). To identify distinct training needs profiles, we performed K-means clustering analysis [26], which groups participants based on their response patterns. Several cluster formations were performed to identify the optimal cluster configuration, which was determined using the silhouette coefficient method [27]. Statistically significant differences between these profiles were analyzed using *t*-tests, Chi-square tests, and Mann–Whitney *U* tests. Detected differences between the two profiles were considered statistically significant when *p* < 0.05. The levels of training needs in nursing students were interpreted as low (Likert scale score < 2.5), intermediate (2.50–3.5), and high (> 3.50).

## Results

### Participant characteristics

**Fig. 1:**
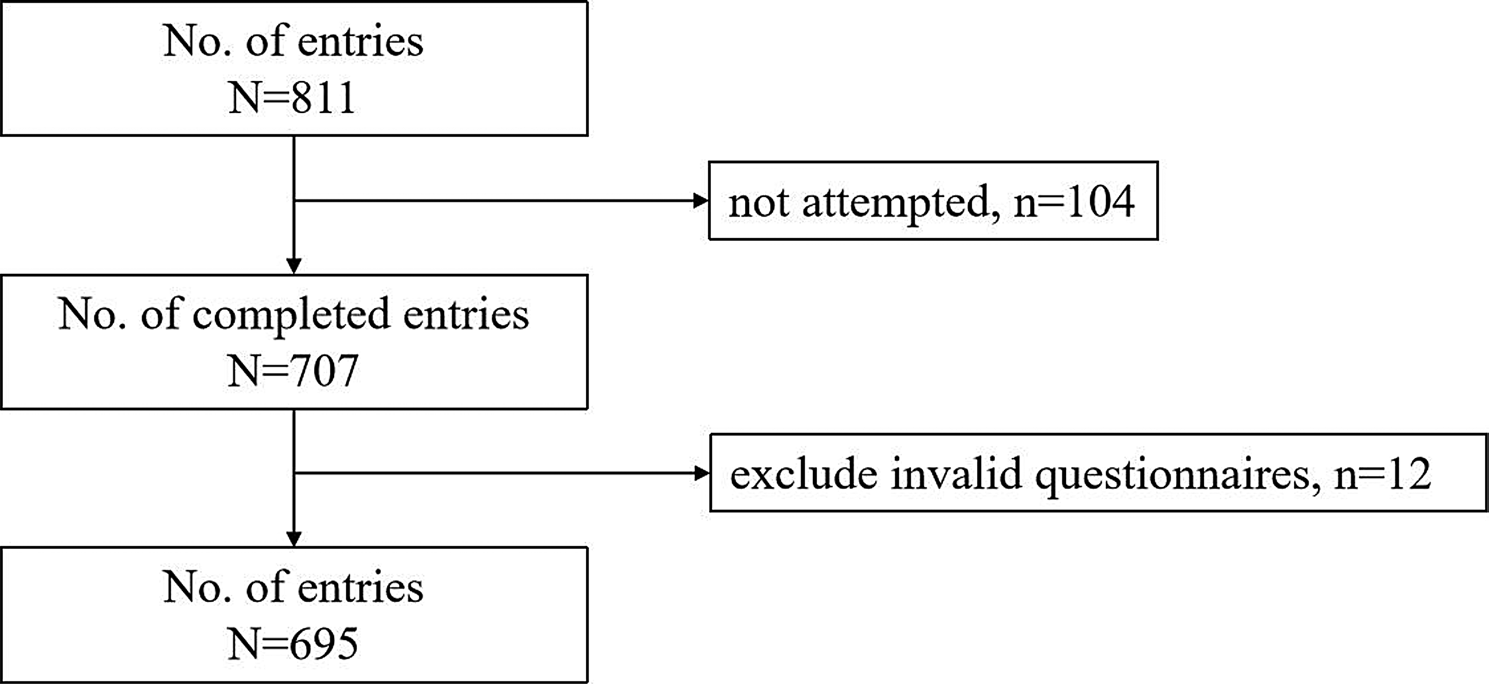
Participant flowchart

The demographic characteristics of the participants are summarized in Table 2. As shown in Fig. 1, a total of 811 undergraduate nursing students from a medical university in southwestern China were invited to take part in the survey. Of these, 707 students responded. After excluding 12 questionnaires deemed invalid due to clearly identical answers, 695 valid questionnaires were analyzed, yielding a valid response rate of 98.3%. As presented in Table 2, the mean age of the participants was 22.40 years (SD: 1.30). The majority was female (*n* = 609, 87.60%), while only a minority had religious beliefs (*n* = 5, 0.70%). Additionally, 27.20% (*n* = 189) was from ethnic minorities, and 56.30% (*n* = 391) was year 3 students. Most participants had no experience in caring for terminally ill patients or their relatives (*n* = 559, 80.40%), had never acquired knowledge about palliative care from nontextbook sources (*n* = 517, 74.40%), and had never participated in palliative care training/seminars (*n* = 510, 73.40%).

**Table 2:** Nursing students’ training needs profiles and characteristics (*N* = 695)

Variables	Total sample	Profile A (*n* = 393)	Profile B (*n* = 302)	*p*-value
Age (years), (mean) (min-max), (SD)	22.40(18–25)(1.30)	22.43(18–25)(1.27)	22.35(19–25)(1.34)	0.535 ^*a*^
Gender (n)(%)				
Male	86(12.4)	55(14.0)	31(10.3)	0.139 ^*b*^
Female	609(87.6)	338(86.0)	271(89.7)	
Ethnicity(n)(%)				
Han	506(72.8)	284(72.3)	222(73.5)	0.715 ^*b*^
Ethnic minorities	189(27.2)	109(27.7)	80(26.5)	
Year level(n)(%)				
Year 3	391(56.3)	221(56.2)	170(56.3)	0.433 ^*b*^
Year 4	304(43.7)	172(43.8)	132(43.7)	
Religious beliefs				
Yes	5(0.7)	4(1.0)	1(0.3)	0.288 ^*b*^
No	690(99.3)	389(99.0)	301(99.7)	
Only child				
Yes	85(12.2)	46(11.7)	39(12.9)	0.630 ^*b*^
No	610(87.8)	347(88.3)	263(87.1)	
Experience in caring for terminally ill patients or their relatives				
Yes	136(19.6)	72(18.3)	64(21.2)	0.344 ^*b*^
No	559(80.4)	321(81.7)	238(78.8)	
Acquisition of knowledge about palliative care from sources non-textbooks				
Yes	178(25.6)	58(14.8)	120(39.7)	**0.000** ^*b*^
No	517(74.4)	335(85.2)	182(60.3)	
Involvement in palliative care training/seminars				
Yes	185(26.6)	104(26.5)	81(26.8)	0.916 ^*b*^
No	510(73.4)	289(73.5)	221(73.2)	

*p*-value < 0.05 marked in bold

a Independent samples t-test. b Chi-square test

### Level of nursing students’ training needs in palliative care education

**Fig. 2:**
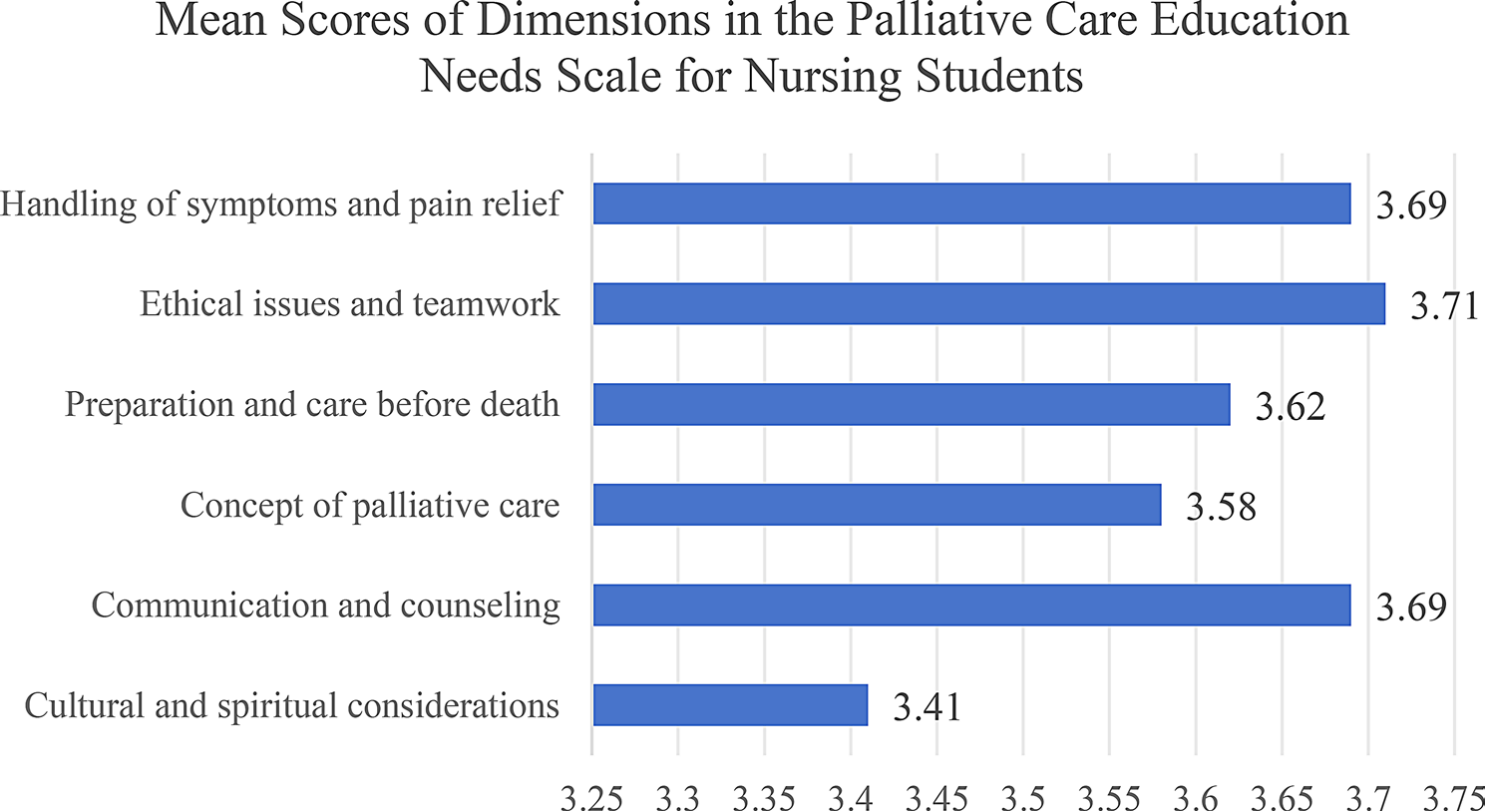
Level of nursing students’ training needs in palliative care education

The educational training needs of nursing students regarding palliative care are at a moderate to high level. The nursing students identified their top training needs as follows: ethical issues and teamwork (mean: 3.71), communication and consultation (mean: 3.69), and managing symptoms and pain relief (mean: 3.69). This was followed by preparation and care before death (mean: 3.62) and concept of palliative care (mean: 3.58). They reported the least training need in the area of cultural and spiritual considerations (mean: 3.41), as shown in Fig. 2.

### Palliative care education: nursing students’ training needs profiles

**Fig. 3:**
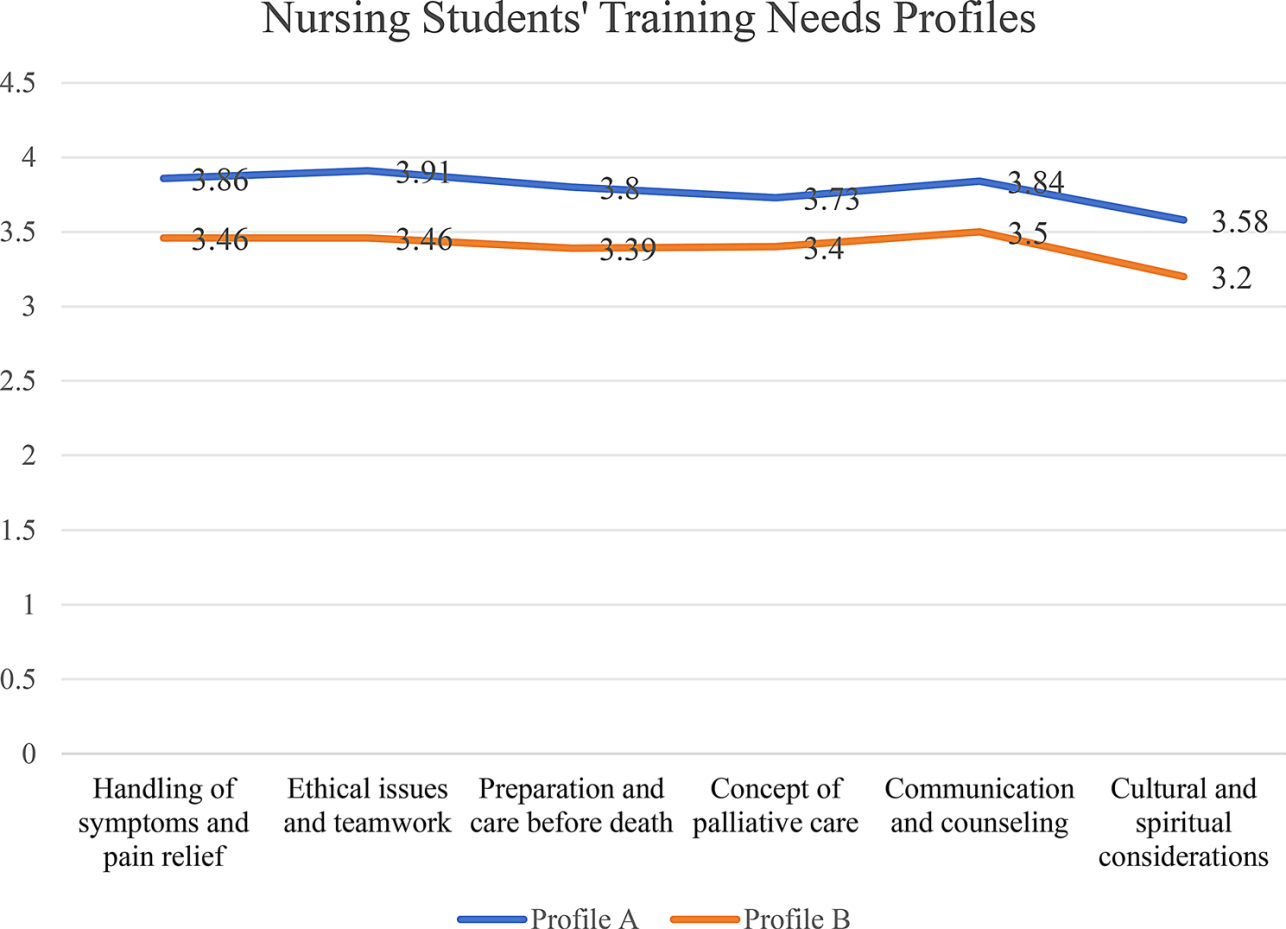
Nursing students’ profiles were categorized based on their training needs in palliative care education

The nursing students were classified into two profiles: those with high-level training needs (mean 3.58 and 3.91) and those with moderate-level needs (mean 3.20 and 3.50) (Fig. 3). Profile A comprised 393 students, 86.0% of whom are female, with an average age of 22. Profile B included 302 students, 89.7% of whom are female, with an average age of 22. Only a minority of students in Profile A (1%) and Profile B (0.3%) has religious beliefs. The differences between profiles in the assessment of training needs were statistically significant in all the areas (*p* < 0.001), as shown in Table 3. Both profiles scored the lowest in “cultural and spiritual considerations” (Profile A mean: 3.58, Profile B mean: 3.20). The largest differences between the profiles were in “ethical issues and teamwork” (Profile A mean: 3.91, Profile B mean: 3.46). The difference in scores for the “concept of palliative care” was the smallest between the profiles (Profile A mean: 3.73, Profile B mean: 3.40). A statistically significant difference was found between the profiles in terms of acquisition of knowledge about palliative care from nontextbook sources (*p* < 0.001). Specifically, 39.7% of nursing students in Profile B had acquired knowledge about palliative care from nontextbook sources, whereas 14.8% of students in Profile A had done so.

**Table 3:** Overview of needs across dimensions in palliative care training for nursing students (*N* = 695)

Sub-dimensions (mean, SD) Likert scale 1–5	Total sample	Profile A (*n* = 393)	Profile B (*n* = 302)	*p*-value	Cohen’s d effect size (*r*)
“Handling of symptoms and pain relief(Q8-15)”	3.69(0.84)	3.86(0.88)	3.46(0.73)	<0.001 ^a^	0.24
“Ethical issues and teamwork(Q33-40)”	3.71(0.80)	3.91(0.86)	3.46(0.64)	<0.001 ^a^	0.28
“Preparation and care before death(Q27-32)”	3.62(0.82)	3.80(0.90)	3.39(0.65)	<0.001 ^a^	0.25
“Concept of palliative care(Q1-7)”	3.58(0.81)	3.73(0.90)	3.40(0.64)	<0.001 ^a^	0.21
“Communication and counseling(Q20-26)”	3.69(0.81)	3.84(0.90)	3.50(0.66)	<0.001 ^a^	0.21
“Cultural and spiritual considerations(Q16-19)”	3.41(0.85)	3.58(0.96)	3.20(0.63)	<0.001 ^a^	0.23

*p*-value < 0.05 marked in bold

a Mann-Whitney U Test

## Discussion

This study assessed palliative care training needs among nursing students and classified them into distinct profiles using K-means clustering. The analysis revealed two clear profiles: Profile A (high needs, *n* = 393) and Profile B (moderate needs, *n* = 302), with significantly different training demands (*p* < 0.001). Both profiles showed high priority needs in *ethical issues and teamwork* (mean = 3.71), *communication and consultation* (3.69), and *managing symptoms and pain relief* (3.69). Notably, 73.4% of participants lacked formal palliative care training, and Profile A students had particularly limited exposure to non-textbook learning. These findings provide an evidence-driven foundation for addressing critical gaps between students’ palliative care training needs and current educational provisions through targeted interventions.

This study identifies two distinct nursing student profiles in palliative care training needs, highlighting the necessity for stratified educational strategies to address their diverse requirements. This strategy aligns with the principles of differentiated instruction, optimizing educational outcomes and learner engagement by adapting to individual needs [28]. The high demand for *ethical issues and teamwork*, *communication and consultation*, and *managing symptoms and pain relief* training across both profiles underscores the essential role of these core competencies in palliative care. Students frequently encounter complex ethical dilemmas (e.g., end-of-life decision-making) and communication challenges (e.g., family conflicts) during clinical rotations [29], which explains the high scores for ethics/teamwork and communication/consultation. However, the current teaching mode dominated by classroom lectures [30] fails to cultivate practical coping abilities, and the high frequency of negative communication experiences among clinical nurses and students, along with the difficulties they encounter when interacting with patients and their families, further highlights this gap [7, 31]. Similarly, symptom management training lacks practical application (e.g., non-pharmacological interventions), leaving students unprepared for real-world challenges [32]. To bridge the gap between theory and practice, high-fidelity simulation should be integrated into the curriculum to allow students to rehearse ethical decision-making and communication skills in realistic scenarios [33, 34]. Evidence indicates that such training markedly improves clinicians’ confidence and competence in managing complex conversations in palliative care settings [33, 34]. Each simulation should be followed by structured feedback and a required reflective exercise [35]. Classroom instruction should employ case-based, interprofessional education that reproduces multidisciplinary decision-making processes, supplemented by regular interprofessional communication workshops [36]. For assessment, dedicated Objective Structured Clinical Examination (OSCE) stations should be implemented to evaluate communication, ethical reasoning, and teamwork, and pre-post measures of student self-efficacy and competence should be used to quantify educational impact [37].

Notably, Both profiles scored lowest on cultural/spiritual considerations (mean < 3.0), This finding echoes the global neglect of spiritual care in healthcare systems [38], highlights the inadequacy of spiritual care content in current palliative care education, and underscores the need to strengthen training in this area. Evidence from Puchalski et al. demonstrates that integrating spiritual care into palliative practice significantly enhances patient quality of life (QoL) scores and reduces end-of-life distress in 78% of cases [39]. Similarly, Balboni et al. report that culturally-competent spiritual care increases patient satisfaction by 30% and lowers hospitalization rates by 25% in the final month of life [40]. These outcomes confirm that cultural and spiritual considerations are foundational to improving patient well-being [41]. By addressing these gaps through targeted training—particularly for Profile A students, who exhibit the most pronounced deficits in these areas—nursing education can better equip students with the competencies necessary to enhance clinical outcomes and deliver holistic patient care in palliative settings.

The higher training needs of Profile A in ‘ethical issues and teamwork’ may reflect their exposure to complex clinical scenarios during rotations, which heightened their awareness of knowledge gaps. This aligns with the findings of González-Pérez et al., who reported that students perceive existing courses as insufficient in practical training (e.g., high-fidelity simulation), hindering their ability to address complex ethical issues [30]. Palliative care nurses often face communication, decision-making, and ethical dilemmas in their daily practice [42]. Teamwork, especially interprofessional teamwork, has become an important pillar in providing high-quality palliative care. However, the training and guidance of nursing students in interprofessional teamwork are insufficient, which makes nursing students lack the necessary skills and knowledge to participate in teams effectively [43]. In terms of symptom management, the current nursing curriculum typically covers basic pain assessment tools and pharmacological interventions (e.g., WHO analgesic ladder) through didactic lectures. The lack of non-pharmacological intervention training (such as symptom cluster management) in the course may lead to students having difficulty dealing with the complex symptoms (e.g., concurrent pain and dyspnea) of patients in clinical practice [44], thereby manifested as a high demand for training. This gap between theoretical knowledge and practical application may explain the high training needs identified in the present study. This finding suggests that nursing educators must urgently develop targeted training programs for Profile A students to address these issues effectively in their future palliative care practice. By contrast, although the difference in scores between the two groups regarding the “concept of palliative care” is relatively small, knowledge of this area among nursing students can still be improved [10]. In addition, the study found that nursing students in Profile B were significantly more likely to acquire palliative care knowledge from non-textbook sources (39.7%) compared to Profile A students (14.8%). This may indicate that informal learning experiences may be a key factor in shaping differences in needs. Profile B students have already developed a substantial foundation in palliative care through autonomous learning [45], potentially reducing their immediate need for formal training. Profile B students’ greater use of alternative learning sources could stem from several factors. Their increased clinical experience, particularly in palliative care settings, may drive them to seek practical knowledge beyond textbooks. These students might also demonstrate more proactive learning behaviors, actively pursuing supplementary materials like workshops or online courses [46]. Additionally, curriculum differences could play a role, with Profile B possibly including students who have taken specialized palliative care electives or participated in simulation-based training [47; 48]. These findings collectively suggest nursing educators should actively promote diversified learning approaches, encouraging students to utilize multiple knowledge resources to expand palliative care competencies [49].

To address these issues and cultivate nursing professionals with high levels of expertise and humanistic care, nursing education institutions need to optimize palliative care educational content and methods continuously. Implementing personalized education within resource-constrained settings requires strategic integration with existing structures. First, palliative care education must be emphasized and incorporated into the core curriculum of nursing education [5], giving special attention to the following areas: ethical considerations and teamwork, communication and counseling skills, cultural sensitivity and spiritual care, and management of symptoms and pain relief. Second, while current palliative care education remains limited, our identified profiles (A/B) enable targeted resource allocation for personalized learning. For Profile A students (high needs), mandatory clinical simulations focusing on ethics and teamwork can be implemented using existing skills labs, supplemented by micro-credentialing in core competencies through short, focused workshops (e.g., 4-hour communication training sessions) [50, 51]. For Profile B students (moderate needs), a blended learning approach combining open-access online modules with peer-led case discussions during routine clinical rotations offers a scalable, resource-efficient solution [35]. This tiered approach uses existing digital tools and peer-led learning to close competency gaps efficiently, requiring no extra platforms or faculty time while maximizing limited resources for impactful education under current constraints. Moreover, the integration of theory and practice should be strengthened by providing students with more practical opportunities, increasing clinical exposure, along with guidance to help them translate their theoretical knowledge into practical skills [7, 9, 52]. Evidence from Brown et al. demonstrates that simulation-based communication training significantly enhances healthcare professionals’ confidence and competence in conducting complex conversations, such as goals-of-care discussions in palliative settings [33]. Similarly, Skedsmo et al. highlight that simulation-based learning in postgraduate nursing education improves clinical decision-making skills, communication abilities, and ethical understanding in palliative care scenarios [34]. By integrating these interactive exercises into the curriculum, students can develop critical clinical decision-making skills and communication competencies essential for palliative care practice, thereby bridging the gap between theoretical knowledge and real-world application [49, 50]. Finally, to ensure the quality and effectiveness of palliative care nursing education, teachers need to engage continually in professional development to be competent in delivering efficient palliative care education [53]. Detailing palliative care content delivery and assessment and evaluation methods clearly is also crucial [54]. A phased implementation strategy is proposed, modeled on an evidence-based 18-hour blended palliative-care module, composed of a 2-hour didactic lecture, 10 h of theory combined with symptom-focused simulation exercises, and 6 h of case‑based simulation practice [55]. In Year 1, core theoretical foundations will be embedded within existing courses. This ensures students establish essential knowledge before advancing to skill-based training. Year 2 focuses on applied learning via intensive case-study analysis and interprofessional simulation, gradually bridging classroom knowledge to clinical practice. By Year 3, tailored elective modules—such as advanced symptom management and psychosocial support—are introduced, following the model of competency-driven customization observed in formative assessments of nursing education. This progressive structure optimizes resource efficiency while accommodating diverse learner needs, ensuring all students achieve core competencies before advancing to specialized training.

Implementing personalized palliative care education in undergraduate nursing faces practical challenges under tight curricular hours. First, learning objectives for undergraduates should focus on four core competencies rather than the full scope required of advanced practice nurses: (a) basic symptom recognition, (b) fundamental communication skills for goals-of-care conversations, (c) ethical awareness in end-of-life decision-making, and (d) interprofessional collaboration awareness. Second, with only limited curricular hours available, we recommend replacing 20% of traditional didactic lectures with “micro-learning” e-modules (≤ 10 min each) [56]. Faculty shortages can be mitigated by training clinical preceptors to provide profile-specific feedback during routine student supervision [57]. Such a scaffolded, competency-focused approach allows gradual integration while remaining realistic for crowded undergraduate curricula.

### Limitation

This study has several limitations. First, due to this study was a single-center study that only investigated nursing students in one university in Southwest China, the results of two profiles(profile A/B) may reflect unique aspects of this particular nursing program rather than representing broader trends. Future multi-site studies could help determine whether these patterns hold across different educational contexts, while qualitative research might explore students’ motivations for selecting various learning resources. While the study’s scope is limited, the validity of the research tools and the representativeness of the results suggest that the findings shed light on general phenomena or trends in palliative care education in Southwest China. Second, the study has common flaws of questionnaire-based studies, such as the objectivity of the results being influenced by the attitude of the participants and insufficient depth of inquiry into the research question. To ensure that the subjectivity of participants did not compromise the objectivity of our findings, we enhanced the response rate by extending the distribution of our questionnaires. This approach allowed us to collect a more representative sample of responses, thereby strengthening the validity of our research outcomes.

## Conclusion

Nursing students exhibit high-level needs of palliative care training, particularly in ethical issues and teamwork, communication and counseling, and handling of symptoms and pain relief. Our findings extend existing literature by identifying two distinct need profiles (high vs. moderate) shaped by prior nontextbook learning exposure, a factor less emphasized in prior research. The findings underscore critical opportunities to enhance palliative care education through targeted curricular innovations and policy reforms. Educational institutions should implement tiered training pathways, prioritizing intensive simulations for high-need learners while reinforcing foundational skills for moderate-need groups. To support this, educational institutions must mandate subgroup-specific competency benchmarks, allocate resources equitably (e.g., expanding simulation access for underserved cohorts), and equip faculty with differentiated instructional tools. Future implementation research should rigorously evaluate the effectiveness of this subgroup-targeted model across diverse regional training centers, assessing its adaptability to varying institutional resources and its long-term impact on clinical competency outcomes.

## Data Availability

The data that support the findings of this study are available from the corresponding author, [RLD], upon reasonable request.
